# Immune response after prolonged hyperoxic mechanical ventilation

**DOI:** 10.1186/cc14328

**Published:** 2015-03-16

**Authors:** HJ Helmerhorst, LR Schouten, NP Juffermans, MJ Schultz, E De Jonge, DJ Van Westerloo

**Affiliations:** 1 Leiden University Medical Center, Leiden, the Netherlands; 2 Academic Medical Center, Amsterdam, the Netherlands

## Introduction

Mechanical ventilation and hyperoxia independently promote pulmonary injury and inflammation. However, the time course of the immune response following concurrent exposure is unclear. The aim of this preclinical study was to study both time-dependent and dose-dependent effects of supplemental oxygen during prolonged ventilatory support on pulmonary inflammation in a well-established murine model of ventilation comparing low and high tidal volumes.

## Methods

Healthy male C57Bl/6J mice, aged 9 to 10 weeks, were randomly assigned to experimental groups (*n *= 8), in which the applied fractions of oxygen (FiO_2_) were 30%, 50% or 90% and tidal volumes were either 7.5 or 15 ml/kg. Anesthetized mice were tracheotomized and ventilated for 8 or 12 hours. Inflammatory cells and mediators were measured in bronchoalveolar lavage fluid (BALf).

## Results

**Figure 1:**
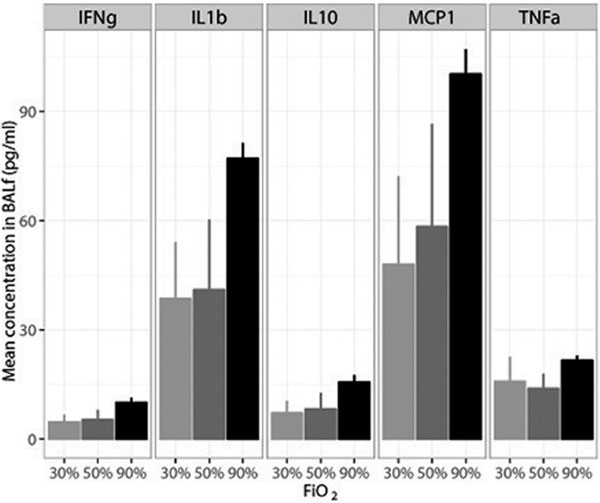
**Inflammatory mediators independent of tidal volumes after 12 hours of MV**.

Mice exposed to higher FiO_2_ had significantly higher PaO_2_ levels at the end of the experiment. The total number of inflammatory cell in the BALf was not significantly different between the experimental groups (*P *= 0.28), yet an increasing trend in the percentage of neutrophils was observed with increasing FiO_2_ (*P *= 0.03). Cytokine and chemokine levels did not differ between FiO_2_ groups at 8 hours of ventilation. In mice ventilated for 12 hours, a significantly increasing trend in IFNγ, IL-1β, IL-10, MCP-1 and TNFα (Figure 1,*P *< 0.01) was measured with increasing FiO_2_, whereas IL-6, KC, MIP-2, GM-CSF and VEGF remained virtually unchanged. Differences between the tidal volume groups were small and did not markedly influence the effects of hyperoxia. See Figure 1.

## Conclusion

Hyperoxia induced a time-dependent and differentiated immune response that was independent of tidal volumes in a model of mechanically ventilated mice. The presence of cytokines and chemokines in the pulmonary compartment was more pronounced with prolonged and severe hyperoxia.

